# Turner Syndrome Mosaicism after Diagnosis of Coeliac Disease—A High Index of Clinical Suspicion Required?

**DOI:** 10.3390/medicina59091693

**Published:** 2023-09-21

**Authors:** F. Ritchie, K. Macgill, D. Cairney, S. Kiff, H. Miles, P. M. Gillett

**Affiliations:** 1 Departments of Paediatric Endocrinology, Royal Hospital for Children and Young People, 50 Little France Crescent, Edinburgh EH16 4TJ, UK; francesca.ritchie@nhs.scot (F.R.); sarah.kiff@nhslothian.scot.nhs.uk (S.K.); harriet.miles@nhslothian.scot.nhs.uk (H.M.); 2 Departments of Gastroenterology, Royal Hospital for Children and Young People, 50 Little France Crescent, Edinburgh EH16 4TJ, UK; kathryn.macgill1@nhs.scot (K.M.); david.cairney@nhs.scot (D.C.)

**Keywords:** coeliac disease, Turner syndrome, Turner mosaic

## Abstract

The association of coeliac disease (CD) in girls with Turner syndrome (TS) is well described. There is, however, a paucity of current research describing TS in patients with known CD. We report two cases of mosaic Turner syndrome diagnosed in girls with CD who failed to achieve expected catch-up growth despite strict adherence to a gluten-free diet (GFD) and the normalisation of TGA-IgA levels. We highlight the need to consider additional diagnoses in patients with CD and ongoing faltering growth. In such patients, referral to a paediatric endocrinologist and relevant investigations, including genetic investigations, should be considered if growth remains suboptimal after one year with a GFD. First-line investigations should include thyroid function, IGF-1, cortisol, gonadotrophins, oestrogen/testosterone, prolactin, karyotype and a bone age X-ray. Clinical suspicion in this situation is key, as an early diagnosis of TS will allow timely treatment with growth hormone, inform discussion around ovarian function and allow screening for important TS associations.

## 1. What Is Known

The association of TS and CD is well described. National recommendations across health care organisations and national expert groups have raised awareness to check for CD in ‘at-risk’ groups, including TS.

## 2. What Is New

Failure to achieve expected catch-up growth in children with CD one year after the introduction of a gluten-free diet should prompt further investigation for an additional pathology, including TS.

## 3. Introduction

We report Turner mosaicism in two females with proven coeliac disease who failed to achieve catch-up growth following adherence to a gluten-free diet.

## 4. Background

Since the late 1990s, there has been increasing research associating Turner syndrome (TS) and coeliac disease (CD) [1], but it has focused on the screening of girls with TS for coeliac disease rather than the suspicion of TS in patients with CD [2,3,4,5,6,7]. The incidence of TS in the general population is estimated to be 1 in 1500 to 1 in 2500 live female births in the UK. A recent Swedish case-control study showed a threefold increased risk of CD in girls with TS compared to the non-CD population. It also confirmed the almost 50% association with other autoimmune diseases and advocated for active case finding [8]. A systematic review highlighted the world literature on TS and CD and suggested that 1 in 22 girls with TS also have coeliac disease [1]. There is a lack of worldwide data on the prevalence of TS in the population initially diagnosed with CD. Two patients presenting to tertiary paediatric services in Edinburgh, UK, are presented below, demonstrating the features of these dual pathologies and relevant investigations.

### 4.1. Case A

Patient A was born at term with a birth weight of 2.65 kg (−2.1 SDS) and a length of 47 cm (second centile) as the first child to non-consanguineous Spanish parents and started weaning at just under 6 months. She presented to paediatric services at the age of 2.4 years with loose watery stools twice a day, bloating and wind, and parental concern about poor growth, her length having tracked below the 0.4th centile. There was also concern related to milk intolerance. Growth measurements at that appointment showed a height of 78.6 cm (−2.5 SDS), a weight of 8.8 kg (−3.8 SDS) and a BMI of 14.2 (−1.88 SDS). No other abnormality was found on examination.

A no-biopsy diagnosis of coeliac disease was made on the basis of TGA-IgA > 10 times the upper limit of normal (153.6 U/mL, NR < 5.0 U/mL). She commenced a gluten-free diet with regular dietetic follow-up. Her TGA-IgA had normalised at 3 months to 4.2 U/mL.

Eight months after her CD diagnosis, her gastrointestinal symptoms had improved. There had been no catch-up growth in weight (9.6 kg, −3.92 SDS) or height (82 cm, −2.77 SDS). She was short compared to her mid-parental height (MPH) of 155 cm (−1.17 SDS).

At her one-year follow-up appointment, this growth pattern persisted despite strict adherence to a GFD. She was phenotypically normal. Additional investigations for short stature were carried out. Her bone age was measured to be 2.76 years (TW2 method) at 3.59 years of chronological age. Her thyroid function, renal function, liver function tests (LFTs) and Insulin-like Growth Factor-1 (IGF-1) were within the normal ranges. Her karyotype revealed a mosaic Turner pattern: 45X (5); 46XX (29). 

**Figure 1:**
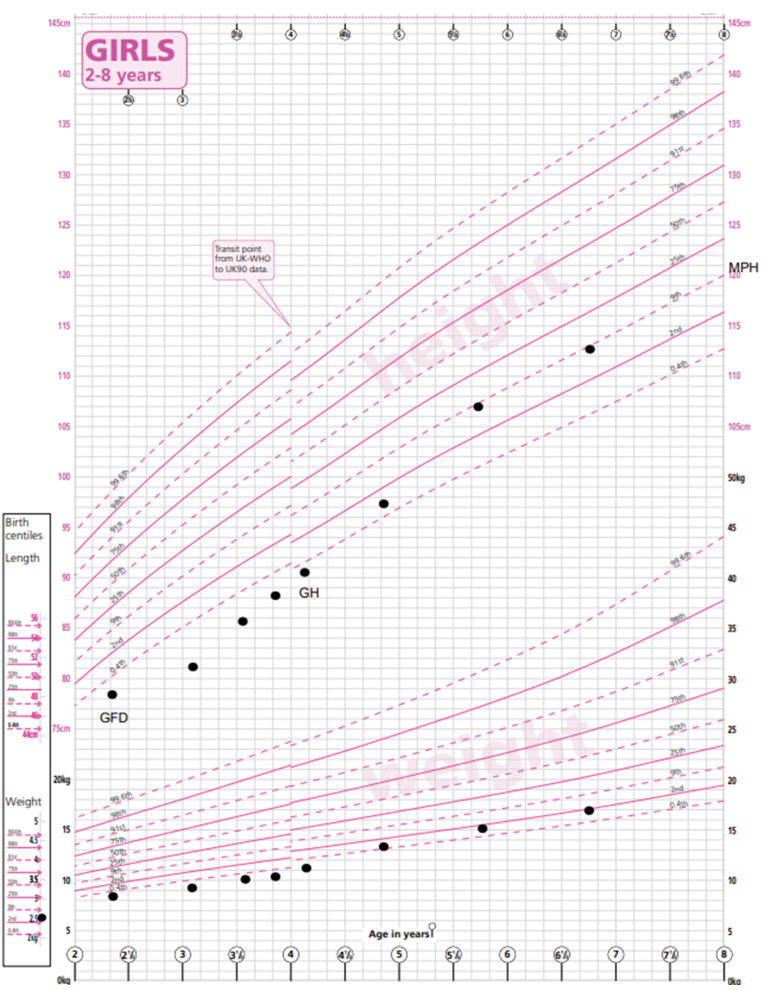
Growth chart depicting height and weight of patient A prior to CD diagnosis and timing of commencing GF diet (GFD) and growth hormone (GH) treatment. Mid-parental height centile (MPH) is indicated. Excellent catch-up growth is demonstrated with introduction of GFD and GH, reaching predicted MPH centile [9]. WHO RCPCH growth charts.

Growth hormone treatment was commenced at 1.4 mg/m^2^ per day via subcutaneous injection as per the recommended dosing schedule for Turner syndrome with a good response in height velocity (9.59 cm/year). Recent measurements at the age of 6.8 years showed a weight of 17 kg (−2.0 SDS), a height of 112.9 cm (−0.98 SDS) and a BMI of 13.3. (−1.65 SDS) (Figure 1). Her height is now appropriate for her mid-parental height. An echocardiogram and a renal ultrasound were performed to exclude other phenotypic manifestations of Turner syndrome and were both normal. Given her mosaic TS genotype with a low proportion of cells with a single X chromosome, ovarian function is difficult to predict. Her baseline gonadotrophins are prepubertal (Luteinising hormone (LH) < 0.5 U/L, Follicle-Stimulating Hormone (FSH) = 2.9 U/L) and appropriate for her current age. Serial Anti-Mullerian Hormone (AMH) measurements have been detectable and stable and will continue to be monitored during and after puberty. A referral to fertility services will be made if appropriate. 

### 4.2. Case B

Patient B was born in the UK to parents of Pakistani origin who are first cousins. She was born at 36 weeks of gestation small for her gestational age with a birth weight of 1.76 kg (−2.4 SDS). 

The patient was referred to paediatric services at 3.3 years of age with abdominal pain, constipation and lethargy. Her weight was 9.67 kg (−4.1 SDS), and her height was 88 cm (−1.83 SDS). Her mid-parental height was 157 cm (−1.58 SDS). Investigations revealed a TGA-IgA > 200 U/mL, and a no-biopsy diagnosis of CD was made. A GFD was commenced. She also had microcytic anaemia due to an iron deficiency and started iron supplementation. Prior to starting a GFD (and iron supplements), she had microcytic anaemia with an Hb level of 109 g/L (115–155), an MCV of 65 fl (77–95) and a ferritin level of 5 ug/L (15–80), and with the commencement of a GFD and a course of iron treatment, her Hb normalised (133 g/L) and has been maintained long term, with normal ferritin levels (22 ug/L). Her thyroid function was normal.

**Figure 2:**
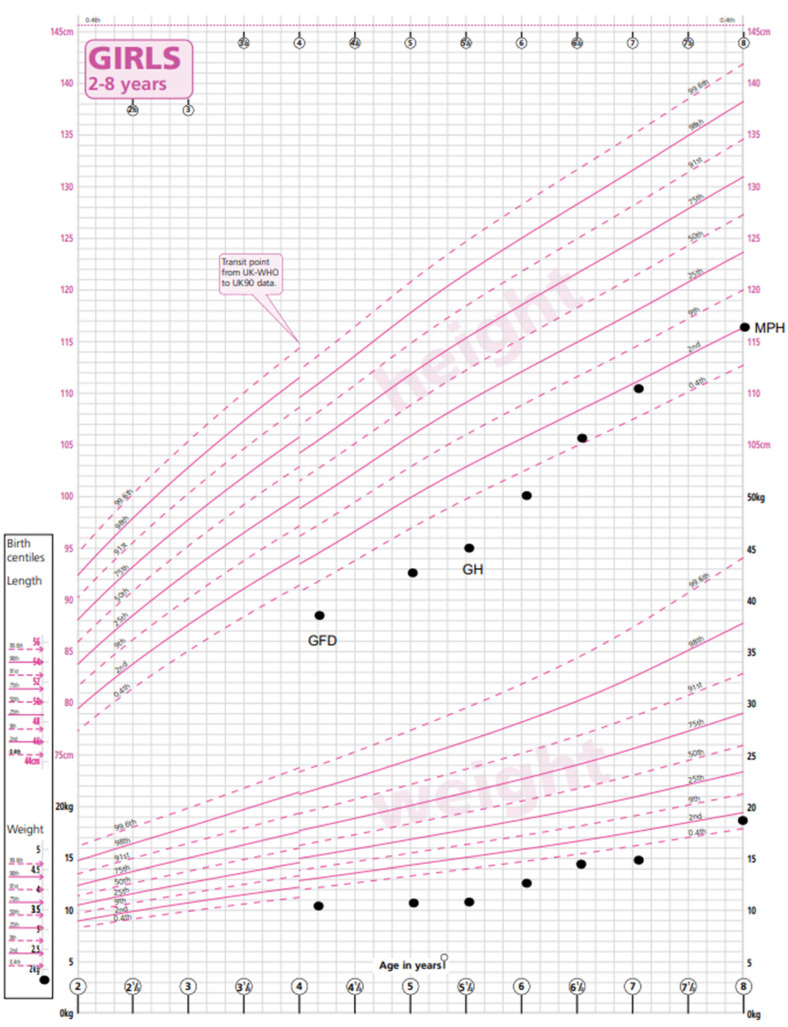
Growth chart depicting height and weight of patient B prior to CD diagnosis and timing of commencing GF diet (GFD) and growth hormone (GH) treatment. Midparental height centile (MPH) is indicated. Excellent catch-up growth is demonstrated with introduction of GFD and GH, reaching predicted MPH centile [9]. WHO RCPCH growth charts.

Within 6 months of commencing a gluten-free diet, patient B’s abdominal symptoms had improved, and by 10 months her TGA-IgA had significantly reduced to 13.4 U/mL, but it took until 20 months for her serology to normalise to 5 U/mL. She had no abnormal phenotypic features. There had been no catch-up growth in height or weight observed, and she was referred to the paediatric endocrine team. A karyotype established a diagnosis of TS with loss of the X chromosome in 40% of cells and partial Xq loss and gain in the remaining 60% of cells. Growth hormone treatment was commenced with a very good effect, and growth measurements at the age of 8.6 years recorded a height of 120 cm (−1.4 SDS, appropriate for her mid-parental height), a weight of 20 kg (−1.87 SDS) and a BMI of 14.4 (−1.05 SDS) (Figure 2).

Screening investigations following the diagnosis of TS found normal renal anatomy on ultrasound and normal cardiac anatomy. AMH was undetectable, indicating primary ovarian insufficiency (POI). Counselling was provided regarding the need for pubertal induction in the future and predicted infertility. Genetic counselling was offered to her parents, who were planning further children.

## 5. Discussion

There is now significant research advising screening for CD in at-risk groups from many health care organisations, including NICE and expert guidelines from national groups, with the incidence of TS in the population between 1 in 1500 and 1 in 2500 live female births [5,7,10]. There is, however, limited guidance around further investigation when growth does not improve upon commencing a gluten-free diet following a diagnosis of coeliac disease. The 2022 ESPGHAN follow-up guideline (Section 5.1) states that the maximum rate of catch-up growth might be expected within the first 6 months of commencing a GFD but it may take 2 to 3 years to achieve a full response. They recommend referral to a paediatric endocrinologist if, after a year, catch-up growth is less than expected [11]. There is little in the literature about secondary diagnoses after a CD diagnosis, especially in this group of patients.

Our two cases did not achieve catch-up growth from their baseline parameters one year post-diagnosis of CD despite strict adherence to a GFD and the stabilisation/normalisation of other parameters, with TGA-IgA normalisation taken as a proxy for mucosal recovery. This triggered clinical suspicion about additional underlying causes of suboptimal growth and prompted referral to the paediatric endocrinology team. In both cases, a diagnosis of mosaic TS was made, allowing GH treatment to be commenced and additional relevant screening tests to be initiated with appropriate management and improved growth. 

Turner syndrome classically presents with a short stature compared to that predicted for the family [12]. Although other phenotypic signs of TS may be present, such as a webbed neck, low-set ears and cubitus valgus, these are not universal, as demonstrated in our cases. Both girls were also born small for their gestational ages (birth weight < 10th centile) and therefore had additional red flags for a second cause of short stature (compared to CD, where poor growth velocity is acquired with the onset of CD).

Growth hormone treatment is well established in TS with evidence that it positively impacts the final adult height [13]. Primary ovarian insufficiency (POI) due to the presence of streak gonads is common, and providing sex steroid replacement therapy is important for girls with TS. Counselling regarding infertility in girls with POI is needed. There is clearly an increased risk of associated autoimmune and cardiovascular diseases in those with TS, and therefore additional screening and vigilance is advised [14,15]. A diagnosis of TS is important to optimise treatment in paediatric and adolescent life. 

When assessing a child or young person with coeliac disease with an ongoing faltering growth pattern despite 1 year with a gluten-free diet, we would recommend taking a history and examining for features of the conditions listed in Table 1.

First-line investigations should specifically include:

- Calculation and plotting of mid-parental height and comparison to current height centile;
- Measurement of height velocity and comparison to normative data (expectation that height velocity should be >50th percentile to result in ‘catch-up’ growth);
- Assessment of pubertal stage;
- Bone age X-ray;
- Biochemistry: thyroid function, gonadotrophins, oestradiol, IGF-1, prolactin, cortisol, renal function, and LFTs;
- Karyotype.

The case should be discussed with or referred to a clinician with expertise in paediatric endocrinology, as additional testing may be warranted depending on these initial results.

These two reported cases highlight the importance of dietitians and gastroenterologists caring for young people with CD being aware of additional causes of growth failure (including TS) and promptly initiating testing or a referral for an appropriate assessment when it has been shown that catch-up growth has not occurred as expected.

## Figures and Tables

**Table 1:** Potential causes of persisting growth failure in patients with coeliac disease. Those conditions most commonly known to have an increased prevalence in the population with CD are highlighted in bold.

**Genetic**	**e.g., Turner syndrome** **Down Syndrome [16]** **Williams Syndrome [17]** Russell Silver Syndrome Noonan Syndrome Skeletal Dysplasias
Endocrine disorders	**Autoimmune hypothyroidism** Growth hormone deficiency **Type 1 diabetes mellitus** Cortisol excess
Chronic diseases	**Non-adherence to GFD in CD** **Inflammatory bowel disease** Cystic fibrosis
Psychosocial deprivation	Including foetal alcohol spectrum disorder
Familial	Constitutional delay in growth and puberty Familial short stature

## Data Availability

Not applicable.

## Abbreviations

Coeliac disease (CD), Anti-Tissue Transglutaminase Antibody (TGA-IgA), Anti-Mullerian Hormone (AMH), Follicle-Stimulating Hormone (FSH), Liver Function Tests (LFTs), Luteinising Hormone (LH), Thyroid-Stimulating Hormone (TSH).
